# T and B Cell Immune Responses to Influenza Viruses in Pigs

**DOI:** 10.3389/fimmu.2019.00098

**Published:** 2019-02-05

**Authors:** Barbara Holzer, Veronica Martini, Matthew Edmans, Elma Tchilian

**Affiliations:** Department of Mucosal Immunology, The Pirbright Institute (BBSRC), Pirbright, United Kingdom

**Keywords:** swine influenza virus (SIV), T cell responses, mucosal immunity, peptide SLA-Tetramer, local T cell immunity, LAIV (live attenuated influenza vaccine), VAERD, neuraminidase

## Abstract

Influenza viruses are an ongoing threat to humans and are endemic in pigs, causing considerable economic losses to farmers. Pigs are also a source of new viruses potentially capable of initiating human pandemics. Many tools including monoclonal antibodies, recombinant cytokines and chemokines, gene probes, tetramers, and inbred pigs allow refined analysis of immune responses against influenza. Recent advances in understanding of the pig innate system indicate that it shares many features with that of humans, although there is a larger gamma delta component. The fine specificity and mechanisms of cross-protective T cell immunity have yet to be fully defined, although it is clear that the local immune response is important. The repertoire of pig antibody response to influenza has not been thoroughly explored. Here we review current understanding of adaptive immune responses against influenza in pigs and the use of the pig as a model to study human disease.

## Introduction

Influenza A viruses (IAV) are a global health threat in humans and animals. Although birds are the major natural reservoirs of IAVs, pigs are a source of novel antigenically distinct IAV, capable of initiating an epidemic or pandemic in humans. Growing demand has made pig farming one of the fastest growing agricultural sectors with yearly productions globally of pigs of nearly a billion animals^1^. IAVs are endemic in the global pig population with H1N1, H1N2, and H3N2 swine influenza viruses (SwIAV) in circulation (1, 2). SwIAV infection in pigs causes a significant economic loss due to reduced weight gain, suboptimal reproductive performance and secondary infections. Pigs can also transmit other diseases. For example acting as a bridge between wildlife (bats) and humans in 1998–1999 in Malaysia and Singapore where a Nipah virus outbreak resulted in the culling of over one million pigs and the deaths of more than 100 people (3, 4).

Effective immunization strategies and biosecurity practices would help eliminate the financial losses due to SwIAV and improve animal welfare. There are several licensed vaccines available for pigs (5), primarily in the United States and these include whole inactivated virus (WIV), an HA subunit vaccine delivered as RNA in an alpha virus vector (6, 7) and a live attenuated influenza vaccine platform (LAIV) (8) in addition to autogenous vaccines. However, these vaccines show poor efficacy in the field because of the rapid evolution of the virus.

In addition to a potential source of new pandemic viruses, the pig is an excellent large animal model of human influenza infection (9, 10). Pigs and humans are infected by the same subtypes of virus and have the same distribution of sialic acid receptors in their respiratory tract (11). The pig is immunologically, physiologically, and anatomically more similar to humans than small animals. Therefore, understanding influenza infection in pigs has enormous potential for combating and controlling IAV in humans.

Despite losses to the pork industry and the potential role of pigs as a source of new IAVs, our understanding of the mechanisms of immunity in pigs lags behind studies in rodents and humans. Here we review the current knowledge of adaptive immune responses following infection and immunization to SwIAV. This has been well-reviewed previously (5, 12–14), so to avoid repetition we concentrated on more recent advances in the field from the last 5 years and areas that have not been covered in the above reviews.

## Antibody Mediated Protection Against SwIAV Infection

### Isotype-Specific Responses After Experimental Infection

Antibodies play a key role in protective immunity to SwIAV and are considered to be the best defined correlate of protection. Antibody responses in WIV immunized pigs focus on the two surface glycoproteins haemagglutinin (HA) and, to a lesser extent, neuraminidase (NA). WIV vaccines induce high titres of neutralizing antibodies that target the immunodominant HA head domain, thereby inhibiting virus entry into host cells. In contrast infection induces much lower levels of neutralizing antibodies whilst still offering protective immunity against homologous, but also heterologous infections (15, 16). A broader antibody response elicited by infection compared to WIV has been noted in both pigs and humans (17–19).

In experimentally-infected pigs the first HA-specific antibodies can be detected as early as 3 days post infection in the serum in some animals although at very low titres (20). The IgM subtype precedes IgG antibodies in the serum, peaking at day seven post infection (dpi). In contrast IgG HA-specific antibodies peak at 25 dpi or later. Secretory IgA in nasal washes is detected as early as 4 days post infection in 50% of experimentally infected pigs (20).

During infection antibody responses against the more conserved internal proteins, such as the nucleoprotein (NP) or matrix proteins M1 and M2, are also mounted. The systemic and mucosal isotype-specific NP antibody responses have been analyzed in experimentally-infected pigs (21). NP-specific antibodies of all three isotypes (IgA, IgM, IgG1) were found in the serum and bronchoalveolar lavage (BAL). In agreement with other reports, IgM peaked first compared to IgA and IgG responses, which were both longer lived. NP-specific IgA and IgM could be detected in the nasal washes by 10 days post infection (dpi) and very low titres of IgG1 appeared only from 30 dpi onwards. Apart from NP-specific responses, antibodies to the M1 and M2 extracellular domain (M2e) could also be detected in the serum of pigs infected with H1N1, H1N2, or H3N2 (22). Only low levels of M2e antibodies were induced by primary infection of naïve pigs, which is in agreement with a previous study that found elevated levels of M2e antibodies in serum only after re-challenge with heterologous SwIAV (15). All isotype-specific and influenza protein-specific antibody responses were determined by ELISA. Potentially non-neutralizing antibodies can contribute to influenza immunity via their Fc function but, on the other hand, have been implicated in vaccine enhanced respiratory disease (VAERD) as discussed below.

There has been increasing interest recently in antibodies against NA elicited by infection or immunization. The sialidase enzymatic activity of NA is responsible for releasing progeny virus from the host cell but is also critical for the transport of virus through mucins (23–25). These important functions of NA in the life cycle of influenza virus makes it an attractive target for vaccine development. There is increasing evidence that links NA antibodies with protection in animal models (26–28) but more importantly also in humans (29–32). Recently a broadly protective mouse monoclonal antibody was described that targets a conserved motif located on the head of the NA influenza B viruses, which offered protection in a mouse model (33).

In pigs there is limited information on NA antibody responses and their role in protective immunity against SwIAV. Sandbulte and colleagues compared the NA inhibitory antibody titres in pigs vaccinated either with intramuscular WIV or intranasal LAIV in comparison to an intranasal wild type infection (34). All three groups had similar levels of serum neuraminidase inhibitory (NI) titres in contrast to the BAL where NI titres were induced only in the LAIV and wild type virus groups. In another study the presence of NI antibodies against the challenge strain correlated with protective immunity in pigs in the absence of conventional neutralizing antibody responses, although additional mediators such as cross-protective T cells may have been involved but were not analyzed (16). In a different study, homologous NA antibodies in the serum were also induced by a temperature sensitive H3N2 LAIV carrying an epitope tag attenuating mutation but not with the same LAIV formulated as a WIV and administered intramuscularly (35). With the increasing evidence for the role of NA antibodies in cross-protection in humans, more studies are required to understand the NA antigenic diversity in SwIV and its importance for vaccine design.

### Mucosal Immunity

The distribution of IgA or IgG antibody-secreting cells (ASCs) specific for influenza was analyzed in variety of tissues in intranasally H1N1-infected pigs (36). Influenza-specific IgG was found in the nasal mucosa (peaking at 21 dpi) with 500 ASCs per million cells, and 10-fold less in the tracheobronchial lymph nodes (TBLN). IgA producing cells were only found in the nasal mucosa, peaking at 14 dpi with up to 3,000 ASCs per million of cells. These data suggest that IgA might be produced locally which was further supported by a study of Heinen and colleagues which compared the influenza specific activity (ratio of NP-specific titer to total Ig concentration) between serum and mucosal sites (21) and found local production of NP-specific IgA and IgG1 in the BAL, and IgA in the nasal mucosa.

Induction of mucosal immunity is an important mediator of protective immunity and WIV vaccines administered intramuscularly are unable to induce a local, mucosal immune response. When NP-specific isotype responses in serum and oral fluids were analyzed in intratracheally challenged pigs that had been immunized with an adjuvanted WIV (Flusure®), IgG NP-specific recall responses were found in the serum of vaccinated pigs. However, no difference in IgA and IgG responses in the oral fluid was detected between immunized and control pigs (37). Additional studies confirmed that live infection is superior to an inactivated vaccine in conferring protection against a drifted H3N2 virus (38). Elevated NP-specific IgA responses in nasal swabs were detected in the live infected pigs at the time of challenge suggesting that induction of mucosal immunity is important for protection and sterilizing immunity.

Intranasal administration of LAIV induces mucosal immunity similar to natural infection in pigs (39, 40). LAIV vaccines carrying either an elastase cleavage site (41), non-structural protein 1 truncations (42), or temperature-sensitive mutations in the polymerase basic protein (PB) 2 and PB1 segments (43, 44) have been shown to be protective in pigs. Heterologous protection was offered by LAIV vaccine candidates in several studies which may be attributed to their ability to induce local IgA responses (45–47) and perhaps local T cell immune responses (see below). In contrast WIVs, which can induce higher titres of neutralizing serum antibodies were not protective or only partially protective, further confirming that using serum haemagglutination titres as a measure of efficacy or cross-protection could be misleading (35, 44).

Secretory IgA responses have been suggested to be a correlate of protection against influenza infections in humans (48–51). In a recent challenge study in healthy adult volunteers, a reduction in number of days of shedding of infectious virus did correlate with higher pre-existing influenza specific nasal or serum IgA (51). Purified IgA from nasal and lungs washes of immunized mice did neutralize homo- and heterologous viruses *in vitro* (52). In contrast to IgG antibodies which only protected against the homologous strain, secretory IgA was less specific and offered cross-protection. Therefore, natural infection or LAIV vaccines administered locally (intranasally or by aerosol) offer better protection than WIV vaccines by inducing immune responses at respiratory mucosal sites.

### Vaccine Associated Enhanced Respiratory Disease

Vaccine associated enhanced respiratory disease (VAERD) has been reported in pigs immunized with WIV followed by a heterologous challenge (53–55). This phenomenon has been reproduced with different SwIAV strains and pigs of different ages, with varied intervals between immunization and challenge.

Although the mechanism responsible for VAERD is not well-understood, it is associated with the presence of high titres of non-neutralizing antibodies targeting the HA2 stalk domain. These promote increased virus infection of Madin–Darby canine kidney (MDCK) cells *in vitro* and enhanced membrane fusion in the absence of neutralizing, anti-head HA antibodies (56). In addition, pro-inflammatory cytokines and cytokine dysregulation were associated with severe lung pathology and neutrophil infiltration. Follow up studies showed that the adjuvant can modulate VAERD and that a temperature-sensitive LAIV vaccine did not induce VAERD after heterologous challenge when compared directly to the WIV vaccine (47, 55). VAERD could be reversed when the NA was matched in the vaccine and challenge strain (57) or dampened when M2 protein was administered in conjunction with the respective WIV (58) suggesting that there is an interplay between antibodies targeting different components of the virus. However, the etiology of VAERD remains controversial as a recent paper showed that induction of VAERD by immunization with an adjuvanted H1N2 vaccine, followed by challenge with pandemic H1N1, did not correlate with the presence of anti-stalk antibodies (59).

Enhanced pathology has not only been reported in the context of WIV. When the nucleoprotein was delivered intramuscularly without adjuvant by virus replicon particles (VRPs) based on vesicular stomatitis virus (VSV) or classical swine fever virus (CSFV) and the pigs were then challenged with a heterologous strain, a higher number of lung lesions were found compared to empty VRPs (60). A DNA-based delivery system encoding a fusion protein of M2e and NP induced severe lung pathology which was associated with antibodies to M2e and a cell mediated response (61). Similarly immunization with HA, M2e and NP targeted to dendritic cell by anti-CD11c antibody exacerbated disease when administered intradermally (62). In contrast intramuscular delivery, without DC targeting, reduced viral shedding and induced a broader antibody response compared to the intradermal route, suggesting that the route of vaccine delivery can also lead to vaccine adverse effects.

### Anti-HA Stalk Antibodies and Fc-Mediated Functions

The HA is composed of two major domains: the immuno-dominant globular head (HA1) domain, that frequently undergoes antigenic drift, and a stalk (HA2) domain, that have been relatively conserved between different influenza virus strains (63). Human seasonal vaccines usually prompt strain specific responses and generate neutralizing monoclonal antibodies (mAbs) to the HA1 domain that prevent virus entry into host cells. These vaccines therefore need constant updating as new antigenic variants emerge, which can no longer be neutralized. In contrast in the last decade many laboratories have described broadly neutralizing antibodies against the conserved stalk which offer protection within and across influenza subtypes (64–75).

There is limited knowledge about the antibody landscape induced by infection in pigs or if broadly neutralizing antibodies can be elicited by sequential exposure to different strains. A recent study analyzed the breadth of the immune response and how it can be modulated by different vaccine regimes in pigs (76). Inactivated vaccines adjuvanted with Emulsigen® from divergent H3N2 lineages were sequentially administered and the immune response to HA and NA determined. The sequential administration of single virus preparations broadened the immune response and produced higher antibody titres to the most divergent viruses than a prime boost regime with the bivalent vaccine formulation. However, neither the heterologous prime boost with single virus vaccine preparations nor any of the other vaccine regimes did increase the titer of anti-stalk antibodies in immunized pigs. The titres ranged between 1:100 and 1:400 in immunized pigs with no significant differences to the mock-vaccinated pigs. These titres are unlikely to mediate protection as baseline titres in human adults are 1:1,600, which are considered to be non-protective (77, 78).

A strategy to induce stalk-specific antibodies is sequential immunization with chimeric HAs consisting of conserved stalk domains with heads from different influenza strains. A recent study used this immunization strategy in pigs to examined stalk-specific responses in the presence of maternal antibodies (59). None of the vaccine approaches induced stalk-specific antibodies which is in contrast to data obtained in mice and ferrets (79–82).

Multiple studies in mice and ferrets have demonstrated that *in vivo* efficacy of broadly cross-neutralizing anti-stalk antibodies is dependent on Fc-dependent mechanism (68, 83, 84) such as phagocytosis (85), antibody-dependent cytotoxicity (ADCC) [reviewed in (86)] or complement-mediated lysis (87–89). Thus, far no reports on monoclonal influenza-specific porcine antibodies have been published therefore the ability of antibodies induced by vaccination or infection to mediate Fc effector functions remains unexplored in the pig model. Therapeutic administration of the human monoclonal, broadly neutralizing FI6 antibody in a pig challenge model showed no efficacy, which was due to lack of binding of human IgG1 to porcine Fc receptors (90). FI6 failed to induce ADCC when porcine PBMCs as effector cells were used, suggesting that Fc mediated functions are an important mechanism of protection of anti-stalk antibodies *in vivo*. However, there is very little information on the distribution of porcine FcR nor are there well-established assays available for analysis of pig Fc-FcR interaction.

## T cell Responses After Infection and Immunization

### The Importance of T cell Immunity in Protection Against Influenza

Protection from IAV-induced symptoms in humans correlates with pre-existing T cell immunity. Early studies showed that cross-reactive CD8 T cells recognizing conserved viral components protected against severe disease (91). More recent studies of the 2009 pandemic also demonstrated that the presence of pre-existing antigen-specific T cells was associated with reduced symptoms and shedding (92, 93). Similarly, recovery from severe H7N9 induced disease is associated with an early robust IFNγ CD8 T cell responses (94). Longer hospitalization is associated with delayed emergence of this population while the high rate of fatality (~40%) was associated with minimal cellular immunity and diminished T cell function.

A long history of experiments in mice has also shown that T cells induced by live virus infection can protect in the absence of neutralizing antibody (13, 95–98). In pigs the broadly protective vaccine candidate, signal minus Flu (S-FLU) reduced lung pathology and viral load in nasal swabs and the lung after homologous and partially matched challenge in the absence of neutralizing antibodies (99). Another study in pigs demonstrated that a cocktail of intranasally-delivered peptide T cell epitopes in degradable nanoparticles induced SwIAV-specific T cells in the lung without generating SwIAV-specific antibodies and reduced viral load in the lung following challenge with a heterologous H1N1 (100). Collectively, these data show the importance of pre-existing T cell immunity in protection from influenza disease.

However, despite the abundance of evidence for the role of CD4 and CD8 T cells in protection against influenza infection in mice and humans, there are few studies in pigs analyzing in depth T cell immunity in response to immunization or infection. Talker and colleagues analyzed the kinetics and magnitude of the influenza specific T cell responses after H1N2 influenza infection in pigs (101). Proliferating Ki67+ CD4 T cells in pigs were detected as early as 4 days post infection (dpi) in the draining TBLN. The highest frequency of triple IFNγ-IL2-TNFα-cytokine producing CD4 cells was in peripheral blood mononuclear cells (PBMC) followed by TBLN with peak frequencies detected at 9 and 12 dpi. At 44 dpi the SwIAV specific response comprised primarily IFNγ single producing cells in the lung, TNFα single producers in the TBLN and triple producers in the blood. Compared to porcine reproductive and respiratory syndrome virus and porcine circovirus type 2, only SwIAV generates a significant increase in CD4 and CD4CD8 cells in the BAL of infected animals suggesting a role for this population during infection (102).

CD8 cells migrated to the lungs and BAL as soon as 6 dpi after challenge with H1N1 virus (103). Similarly, in H1N2-infected pigs, activated CD27+Ki67+ CD8 T cells peaked at 6 dpi in the lung and they were able to produce both IFNγ alone or in combination with TNFα as well as secrete perforin up to 44 dpi (101). That kinetic is resembled in humans, where IAV-specific T cells peaked 7 days after H1N1 infection with contraction at 4 weeks in PBMC (104).

### Specificity of Immune Response and Peptide Tetramers

The lack in understanding of porcine immune responses has been due, at least in part, to a lack of research tools to study T cell responses in pigs and inability to culture pig T cells *in vitro*. Immunological tools such as robust MHC (in pigs called swine leucocyte antigens, SLA) peptide binding motifs, defined T cell epitopes and peptide-MHC multimer technology for pigs have lagged behind that available in humans or experimental mice. However, putative SwIAV epitopes, restricted by one of the most commonly occurring SLA in outbred pigs, SLA-1^*^0401 (105) or SLA-1^*^0702 (106) were identified using an *in silico* predictive algorithm (Table 1). Another study, using the immunoinformatics tool PigMatrix, identified a number of SLA-1 epitopes highly conserved in seven representative SwIAV strains in the US (108).

**Table 1:** T cell epitopes recognized after immunization or infectious challenge in pigs.

**Epitope sequence**	**Viral protein**	**Virus of origin**	**% of tetramer + cells in tissues^†^**	**SLA**	**References**
DFEREGYSL	NP	A/PuertoRico/8/1934 (H1N1)	0.023–0.03 PBMC 0.045–0.052 TBLN 0.091–6.48 BAL	SLA-1^*^14:02	(107)
EFEDLTFLA	NP	A/PuertoRico/8/1934 (H1N1)	0.014–0.017 PBMC 0.039–0.047 TBLN 0.078–0.17 BAL	SLA-1^*^14:02	(107)
IAYERMCNI	NP	A/PuertoRico/8/1934 (H1N1)	0.1–0.13 PBMC 0.22–0.36TBLN 4.63–11.4 BAL	SLA-2^*^11:04	(107)
NGKWMRELI	NP	A/PuertoRico/8/1934 (H1N1)	0.047–0.036 PBMC 0.037–0.086 TBLN 3.02–11.9 BAL	SLA-2^*^11:04	(107)
SLSTASSWSY	HA	A/swine/Denmark/101310-1/2011 (H1N1)	1.2–1.9 PBMC	SLA-1^*^0702	(106)
TLYQNNHTY	HA	A/swine/Spain/SF11131/2007 (H1N1)	1 PBMC	SLA-1^*^0702	(106)
YVSVGSSKY	HA	A/swine/Spain/SF11131/2007 (H1N1)	0.4 PBMC	SLA-1^*^0702	(106)
CPVSGWAIY	NA	A/swine/Denmark/101310-1/2011 (H1N1)	1.7 PBMC	SLA-1^*^0702	(106)
CPIGEVPSPY	NA	A/swine/Denmark/101310-1/2011 (H1N1)	0.7–2.6 PBMC	SLA-1^*^0702	(106)
GPSNGQASY	NA	A/swine/Denmark/101310-1/2011 (H1N1)	2.1 PBMC	SLA-1^*^0702	(106)
EMNAPNYHY	NA	A/swine/Denmark/101310-1/2011 (H1N1)	0.4 PBMC	SLA-1^*^0702	(106)
NMDRAVKLY	M1	A/swine/Denmark/101310-1/2011 (H1N1)	0.7 PBMC	SLA-1^*^0702	(106)
ALASCMGLIY	M1	A/swine/Denmark/101310-1/2011 (H1N1)	0.7–1.2 PBMC	SLA-1^*^0702	(106)
LASCMGLIY	M1	A/swine/Denmark/101310-1/2011(H1N1)	0.7 PBMC	SLA-1^*^0702	(106)
CTELKLSDY	NP	A/swine/Denmark/101310-1/2011(H1N1pdm09) A/swine/Denmark/ 101568-1/2011 (H1N2) A/swine/Denmark/ 19126/1993 (H1N1) A/swine/Denmark/ 101490-3/2011 (H1N1) A/swine/Denmark/1037- 2/2011 (H1N2)	1.7–6.3 PBMC	SLA-1^*^0401	(105)
GTEKLTITY	PB2	A/swine/Denmark/101310-1/2011(H1N1pdm09) A/swine/Denmark/ 101568-1/2011 (H1N2) A/swine/Denmark/ 19126/1993 (H1N1) A/swine/Denmark/ 101490-3/2011 (H1N1) A/swine/Denmark/1037- 2/2011 (H1N2)	1.5–6.5 PBMC	SLA-1^*^0401	(105)
SSSFSFGGF	PB2	A/swine/Denmark/101310-1/2011(H1N1pdm09) A/swine/Denmark/101568-1/2011 (H1N2) A/swine/Denmark/ 19126/1993 (H1N1) A/swine/Denmark/ 101490-3/2011 (H1N1) A/swine/Denmark/1037- 2/2011 (H1N2)	1.4–4.9 PBMC	SLA-1^*^0401	(105)
YVFVGTSRY	HA	A/swine/Denmark/101310-1/2011(H1N1pdm09) A/swine/Denmark/ 101568-1/2011 (H1N2) A/swine/Denmark/ 19126/1993 (H1N1) A/swine/Denmark/ 101490-3/2011 (H1N1) A/swine/Denmark/1037- 2/2011 (H1N2)	1.5-5.9 PBMC	SLA-1^*^0401	(105)

† *Minimum and maximum percentage of tetramer+CD3+CD8+ cells in blood (PBMC), tracheobronchial lymph nodes (TBLN) and bronchoalveolar lavage (BAL) are given. Only percentages at least double the background or negative control peptide responses are included*.

A recent study in the Babraham large white, inbred pig that is 85% identical by genome wide SNP analysis (109), further developed the porcine immunological toolbox (107). For the first time long-term *in vitro* pig T cell culture and cloning was developed which allowed the identification of novel immunodominant influenza-derived T cell epitopes. Structures of the two SLA class I molecules expressed in Babrahams presenting the immunodominant epitopes were generated. These structures allowed definition of the primary anchors for epitopes in the SLA binding groove and established SLA binding motifs which were used to predict successfully other influenza-derived peptide sequences capable of stimulating T-cells. Peptide-SLA tetramers were constructed and used to track influenza-specific T-cells *ex vivo* in blood, lungs, and draining lymph nodes. Four different NP peptides derived from influenza PR8 virus were confirmed to bind to SLA-1^*^14:02 and SLA-2^*^11:04 (107) (Table 1). The matching of SLA class I and II alleles between individual animals makes the Babraham pigs invaluable for immunological studies, allowing adoptive transfer of immune cells between individuals.

### Live Attenuated Influenza Vaccine-Induced T cell Responses and Local T cell Immunity

Experimental LAIV have been tested extensively in pigs with promising results and attenuated influenza vaccine platform has recently been approved for use in the USA (8). A recent study compared WIV and LAIV vaccines for protection against challenge with antigenically distinct H3N2 viruses in pigs (35). WIV provided partial protection against antigenically distinct viruses but did not prevent virus replication in the upper respiratory tract. In contrast LAIV consistently conferred efficient protection against matched and mismatched strains, which is in agreement with other studies that have compared the vaccine efficacy of WIV and LAIV in pigs (44, 110). Cellular responses were not compared across the two platforms in this study. However, previous work in pigs demonstrated that LAIV can induce a T cell response in naïve pigs (42, 111, 112) which is more robust compared to those adjuvanted WIV immunized pig (5, 112).

Another LAIV is the broadly protective vaccine candidate S-FLU, which is limited to a single cycle of replication through the inactivation of the HA signal sequence (113). S-FLU induces a strong cross-reactive T cell response, but minimal Ab response to the HA after aerosol or intranasal administration (99, 113, 114). Babrahams pigs immunized with S-FLU by aerosol recognized four different NP peptides, three of which were recognized by up to 40% of CD8 T cells present in the BAL 28 days after the boost (107). In pigs pulmonary immunization with S-FLU reduced viral load in nasal swabs and lung after challenge with homologous or partially matched virus (99). However, after heterosubtypic challenge S-FLU reduced lung pathology but not viral load (115). In contrast in ferrets the same S-FLU reduced viral replication and aerosol transmission, suggesting that there are differences in protection between small and large animals.

Overall these studies indicate that LAIV can provide broader protection most likely due to the induction of local lung immune responses and tissue resident memory cells (TRM) which play a major role in protection against influenza (116, 117). The recently discovered lung TRM (118) reside in the respiratory tract without recirculating, unlike central or effector memory T cells, therefore constituting a first line of adaptive cellular defense. Different roles have been attributed to TRM (119): (i) cytotoxicity mediated by constitutive granzyme B expression, (ii) secretion of IFNγ and recruitment of immune cells, (iii) activation of NK cells and dendritic cells, and (iv) upregulation of innate immune components. Pulmonary TRM in the BAL and lung tissues have greater protective capacity than circulating memory CD8 T cells (116, 120). In mice TRM are indispensable for protection against heterologous IAV (116) and vaccines targeting TRM are required to establish heterosubtypic immunity (117). CD4 and CD8 TRM in the respiratory tract express the markers of residency CD103 and CD69.

TRM were recently identified in the lung of S-FLU aerosol immunized pigs following intravenous administration of CD3 antibody (115). More than 90% of BAL cells were inaccessible to intravascular Ab as well as a proportion of the lung tissue cells, but their phenotype and function has yet to be fully established. Aerosol immunization with H3N2 S-FLU induced a strong immune response of these cells (115), which may be able to reduce local inflammation through the release of immuno-modulating cytokines (121, 122).

The newly approved pig LAIV vaccine^2^, may provide broader protection than traditional WIV. As in humans the increased vaccine-induced local T cell and/or secretory IgA response may explain its higher efficacy. LAIV for humans showed decreased efficacy during the 2016–2017 vaccination season in the US, although it continued to show high efficacy in the UK (123). It will be interesting to see if LAIV will show reduced efficacy over time in pigs and whether increased baseline immunity may interfere with vaccine-virus replication.

## Conclusions and Future Challenges

Despite the economic importance of SwIAV and the use of vaccines, the mechanisms of immunity to SwIAV in pigs have been less well-studied, compared to rodents and humans. Further understanding of innate and adaptive immune responses and the way in which they interact is an urgent priority. Results with LAIV vaccines suggest that these may be effective in pigs as in humans, and that this may be because local immunity is induced, but much further work to define local mechanisms and determine optimum means of invoking these responses by immunization, is needed. The discovery of broadly-neutralizing antibodies in humans following infection with IAV and immunization suggests that broadly cross-protective immunity can be achieved by antibody mediated as well as T cell mechanisms. However, there is little published information on broadly neutralizing antibodies in pigs and only fragmentary data on the specificity of cross protective T cells. Similarly, although Fc-FcR interactions are likely to be important in both antibody-mediated *in vivo* protection and VAERD, these effector mechanisms are ill-defined, as is the importance of IgA antibodies. Overcoming these challenges will throw new light on immune responses to influenza viruses and provide information on how best to construct and administer influenza vaccines to provide “universal” protection in pigs and humans.

## Author Contributions

VM wrote the section on T cell responses. ME wrote the section on innate immune responses in the original manuscript and proofread. BH wrote the section on antibody responses and proofread. ET wrote the sections Introduction and Conclusions, reviewed and edited all other parts of the manuscript.

### Conflict of Interest Statement

The authors declare that the research was conducted in the absence of any commercial or financial relationships that could be construed as a potential conflict of interest.

## Footnotes

^1^Food and Agriculture Organization of the United Nations, Food and Agriculture data, Production of pigs in the world. Available online at: http://www.fao.org/faostat/en/#home (Accessed December 20, 2018).

^2^Ingelvac Provenza™, website: https://www.bi-vetmedica.com/species/swine/products/Ingelvac-provenza?gclid=Cj0KCQjw6fvdBRCbARIsABGZvSynUcdx94ljnN9E4XIyrHcLD6EuT7jyh27gONm4m9aUpHzf5TurwYaAmIPEALw_wcB">products/Ingelvac-provenza?gclid=Cj0KCQjw6fvdBRCbARIsABGZvSynUcdx94ljnN9E4XIyrHcLD6EuT7jyh27gONm4m9aUpHzf5TurwYaAmIPEALw_wcB">https://www.bi-vetmedica.com/species/swine/products/Ingelvac-provenza?gclid=Cj0KCQjw6fvdBRCbARIsABGZvSynUcdx94ljnN9E4XIyrHcLD6EuT7jyh27gONm4m9aUpHzf5TurwYaAmIPEALw_wcB">https://www.bi-vetmedica.com/species/swine/products/Ingelvac-provenza?gclid=Cj0KCQjw6fvdBRCbARIsABGZvSynUcdx94ljnN9E4XIyrHcLD6EuT7jyh27gONm4m9aUpHzf5TurwYaAmIPEALw_wcB">products/Ingelvac-provenza?gclid=Cj0KCQjw6fvdBRCbARIsABGZvSynUcdx94ljnN9E4XIyrHcLD6EuT7jyh27gONm4m9aUpHzf5TurwYaAmIPEALw_wcB
